# Dynamic Displays Enhance the Ability to Discriminate Genuine and Posed Facial Expressions of Emotion

**DOI:** 10.3389/fpsyg.2018.00672

**Published:** 2018-05-29

**Authors:** Shushi Namba, Russell S. Kabir, Makoto Miyatani, Takashi Nakao

**Affiliations:** ^1^Graduate School of Education, Hiroshima University, Hiroshima, Japan; ^2^Department of Psychology, Hiroshima University, Hiroshima, Japan

**Keywords:** spontaneous facial expressions, posed facial expressions, dynamics, facial expressions, emotion

## Abstract

Accurately gauging the emotional experience of another person is important for navigating interpersonal interactions. This study investigated whether perceivers are capable of distinguishing between unintentionally expressed (genuine) and intentionally manipulated (posed) facial expressions attributed to four major emotions: amusement, disgust, sadness, and surprise. Sensitivity to this discrimination was explored by comparing unstaged dynamic and static facial stimuli and analyzing the results with signal detection theory. Participants indicated whether facial stimuli presented on a screen depicted a person *showing* a given emotion and whether that person was *feeling* a given emotion. The results showed that genuine displays were evaluated more as felt expressions than posed displays for all target emotions presented. In addition, sensitivity to the perception of emotional experience, or discriminability, was enhanced in dynamic facial displays, but was less pronounced in the case of static displays. This finding indicates that dynamic information in facial displays contributes to the ability to accurately infer the emotional experiences of another person.

## Introduction

Facial expressions provide a signature of the emotional state of an interlocutor to indicate behaviors that are appropriate in an interpersonal situation (Keltner and Haidt, 2001; Ekman, 2003). However, not all facial displays reflect emotional experiences that are actually being felt by the expresser, and can even be co-opted. Humans have been shown to be able to feign facial expressions of felt emotions as a form of intentional deception to gain social advantages (Krumhuber and Manstead, 2009) and to stage displays that are meant to solicit the help of others (Ekman, 2001). Staged or posed facial expressions display an emotion that an expresser ostensibly intends to convey, whereas unstaged or genuine expressions are thought to portend the sense of authenticity that accompanies the spontaneity of felt emotional expressions. The endogenous nature of emotional experiences is posited to increase the trustworthiness of the expresser by emboldening the need to embark upon and ensure a successful social interaction. For example, Johnston et al. (2010) showed that genuine smiles could make perceivers opt for cooperative behavior more than posed smiles. On the other side of the spectrum, pretending to be sad is an expressive strategy that leads to loss consequences for the perceiver when an expresser feigns sadness to take advantage of a perceiver’s reciprocal kindness or compensatory behavior in response (Reed and DeScioli, 2017). Thus, the ability to differentiate genuine displays of emotional experiences from posed ones can be important for dealing with day-to-day social interactions.

Recent work has been conducted on whether people can distinguish between genuine and posed displays of emotion (e.g., McLellan et al., 2010; Douglas et al., 2012; Dawel et al., 2015). McLellan et al. (2010) showed that adults are capable of differentiating posed and genuine facial displays of happiness, sadness, and fear. Dawel et al. (2015) also replicated the finding that adults could discriminate the authenticity of happy and sad displays. Moreover, a neuroimaging study showed that the perception of genuine and posed non-verbal behaviors occurs through different neural activation processes (McLellan et al., 2012; McGettigan et al., 2013). Although there have been few studies that investigate this ability, most prior research suggests that people can make a distinction when judging genuine and posed facial displays.

Nevertheless, previous research has suffered from two major shortcomings: (1) the presence of “staged” contamination in genuine displays due to a lack of accounting for the possible effects of intentional manipulation, and (2) a failure to include dynamic aspects when preparing facial stimuli for experimental investigations. First, research methodologies have mainly relied upon the proprietary facial stimuli created by McLellan et al. (2010), which recruited participants who were expressly informed of the purpose of the study as one to investigate the feasibility of creating stimulus material. The experimenters then proceeded recording the facial expressions of participants as they were evoked by emotion elicitation pictures, sounds, and imagined scenarios. While the experimenters selected genuine displays based on databases of affective picture stimuli and other established experimental techniques from empirical studies, the fact that participants were made aware of the purpose of the facial stimuli ahead of the experiment might have allowed for the confounding effects of intentional manipulation to occur in genuine facial displays as they unfolded. This raises an issue as it is thought that such intentional influences might inhibit spontaneous facial reactions (Smoski and Bachorowski, 2003; Kunzmann et al., 2005). Furthermore, selection of genuine stimuli in the study relied heavily on criteria undertaken for intended facial expressions made by actors (Gosselin et al., 1995; Suzuki and Naitoh, 2003), as several findings have shown actors’ expressions to be relatively similar to spontaneous expressions (e.g., Carroll and Russell, 1997; Scherer and Ellgring, 2007; Gosselin et al., 2010). While it is indeed the case that expressions made by professional actors might encompass some experiences of felt emotion in the process, they are ultimately designed to emphasize a message through intentional or strategic manipulation (Buck and VanLear, 2002). This suggests that facial stimuli used in previous studies could have been biased from being subject to intentional manipulation by participants themselves or through selection criteria that was based on the staged facial expressions of actors. Indeed, McLellan et al. (2010) tagged the cheek raising found in the expression of happiness as a property that distinguishes genuine and posed smiles, but other studies have shown that the presence of cheek raising more likely reflects expressive intensity rather than pleasant experience (Krumhuber and Manstead, 2009; Guo et al., 2018). In other words, previous studies might actually be tapping differences in expressive intensity rather than an underlying ability to tell the difference between posed versus genuine expressions. Recent work by Dawel et al. (2017) also showed that observers did not regard the McLellan et al. (2010) genuine faces as actual genuine displays. Thus, it is clear that to better understand the ability for individuals to differentiate genuine displays containing emotional experiences from posed ones, unintentionally manipulated displays that are most frequently expressed in strong evocations of genuine emotional situations should be implemented.

Second, previous experiments have employed static facial stimuli and largely ignored the dynamic aspects of facial expressions. Dynamic information in facial expressions for various emotions has been increasingly recognized as an important aspect in the phenomenon of emotion perception (Krumhuber et al., 2013) and the recognition of crowd valence (Ceccarini and Caudek, 2013). Ceccarini and Caudek (2013) found that dynamic over static facial information captures the attention of perceivers attending to threatening stimuli. Furthermore, Krumhuber and Manstead (2009) showed that observers can differentiate spontaneous and posed smiles when rating the genuineness and amusement of dynamic displays, but not static ones. Although the importance of dynamic information in differentiating facial expressions has been put forth, not all emotions have been accounted for. Given the evidence from previous studies that have underscored the dynamic aspects of facial expression for emotion perception (e.g., Wehrle et al., 2000; Sato and Yoshikawa, 2007), operationalizing dynamic displays as stimuli for other emotions like surprise, disgust, and sadness, in addition to amusement, would allow for sensitivity in the perception of emotional experience to be evaluated. Taken together, it remains unclear whether people can differentiate genuine from posed facial displays because there is a possibility that the genuine displays used in previous studies are different from spontaneous facial reactions to emotional experiences. Moreover, it is necessary to consider dynamic information that might affect this discriminability beyond the emotion of amusement through investigations of other emotions like surprise, disgust, and sadness.

Thus, the current study re-investigated hypotheses related to the ability for perceivers to distinguish genuine from posed facial expressions by critically implementing facial display stimuli generated in the absence of intentional manipulation. This effort aimed to eliminate the influence of intentional effects in genuine facial stimuli as much as possible to test the assumption in the literature that people can differentiate between genuine and posed facial expressions (McLellan et al., 2010; Douglas et al., 2012; Dawel et al., 2015). Furthermore, this study explored whether the presence of dynamic information in facial stimuli strengthens this genuine-posed discriminability or not in the case of negative emotions in addition to amusement. Considering the findings of Krumhuber and Manstead (2009), it was assumed that sensitivity to this discrimination would be increased for dynamic displays as compared to static ones, and that the evidence base for the phenomenon would be extended beyond amusement to surprise, disgust, and sadness. To further control for the effects of expressive intent as much as possible, the current study utilized the spontaneous facial data obtained in a previous study (Namba et al., 2017a). Spontaneous and posed facial expressions for the emotions of amusement, disgust, surprise, and sadness were recorded to compare morphological aspects in that study, where video clips of secretly recorded facial behaviors as expressers experienced a strong emotion in a room by themselves were used as genuine displays. Posed facial stimuli were derived from the same data of expressers intentionally generating facial expressions according to explicit instructions (for further detail, see Namba et al., 2017c).

## Materials and Methods

### Participants

Fifty-eight participants (35 female, 23 male; *M* age = 23.98, *SD* = 1.67) were recruited from Hiroshima University and the local community via e-mail and advertisements, and were compensated with 500 yen after the experiment. Participants were randomly assigned to one of two groups: (a) *dynamic presentation* (12 female, 18 male; *M* age = 24.00, *SD* = 1.49), and (b) *static presentation* (11 female, 17 male; *M* age = 23.96, *SD* = 1.86). This assignment resulted in 30 individuals designated to the *dynamic presentation* group, and 28 individuals designated to the *static presentation* group. All participants were native Japanese speakers with normal or corrected-to-normal vision. There was no evidence of the presence of a neurological or psychiatric disorder. Written informed consent was obtained from each participant before the investigation, in line with a protocol approved by the Ethical Committee of the Graduate School of Education, Hiroshima University.

### Stimuli

Clips of spontaneous and posed facial actions induced without expressive intent recorded in Namba et al. (2017c) were used as genuine and posed facial displays. Genuine facial displays were elicited in an individual environment with emotion elicitation films (Gross and Levenson, 1995), while posed facial displays were expressed in accordance with the explicit instruction “to express the target emotion.” Namba et al. (2017c) picked only the four emotion types of amusement, surprise, disgust and sadness that were confirmed by a previous study to elicit target emotions in Japanese adults viewing emotion elicitation films (Sato et al., 2007). After recording their genuine expressions, participants were debriefed about their candid recordings in line with protocols set by the Ethical Committee of the Graduate School of Education, Hiroshima University, to which data collection was affirmed or denied if the participant consented to the use of their recordings for analysis, and in the event that consent was not given, the recorded data was deleted in front of the participant (Namba et al., 2017c). Among these facial displays, the parts of the clips to be used as stimuli were selected based on the following criteria: (1) the spontaneous and posed facial expressions contained the most frequently expressed and representative properties among expressers (Namba et al., 2017c), (2) the spontaneous facial expression contained facial components related to target emotional experiences in other empirical studies (Namba et al., 2017a,b), and (3) the same expresser was present in both the spontaneous and posed facial expressions in order to avoid inter-target differences. Additionally, dynamic and static presentations were created using these clips. In dynamic presentations, facial displays were played continuously from onset to peak display of facial expression. In static presentations, facial displays were edited such that only one peak frame of a facial expression was presented. Two expressers were assigned to each emotion including a neutral state representing no emotion. Consequently, 2 (expresser) × 4 (emotion: amusement, disgust, surprise and sadness) × 3 (display: genuine, posed and neutral) × 2 (presentation style: dynamic and static) clips were used, resulting in 48 total clips and 24 clips per presentation style. For dynamic presentation, the mean duration of unfolding genuine facial displays was 2.88 s (*SD* = 2.03), whereas those of posed and neutral ones were 2.50 and 2.38 s (*SD*s = 1.07 and 1.30). Welch’s two sample *t*-test revealed that the durations among all displays were not different (uncorrected *p*s > 0.57). The overall durations were 2.58 s (*SD* = 1.47), and for static presentation all durations were set to 2.5 s. Furthermore, we checked the perceived intensity of expressions as a preliminary analysis. Seven individuals (3 female, 4 male) evaluated the intensity of facial clips on an 8-point scale ranging from 0 (*not at all*) to 7 (*the strongest*). One-way analysis of variance revealed that the perceived intensity was different among three displays [*F*(2,110) = 128.69, *p* < 0.001]. Multiple comparisons also showed differences between neutral (*M* = 0.41, *SD* = 0.61) and genuine (*M* = 3.52, *SD* = 2.13) or posed (*M* = 3.88, *SD* = 1.81; *p*s < 0.001), but no significant difference was found between genuine and posed displays (*p* = 0.08).

### Procedure

**FIGURE 1:**
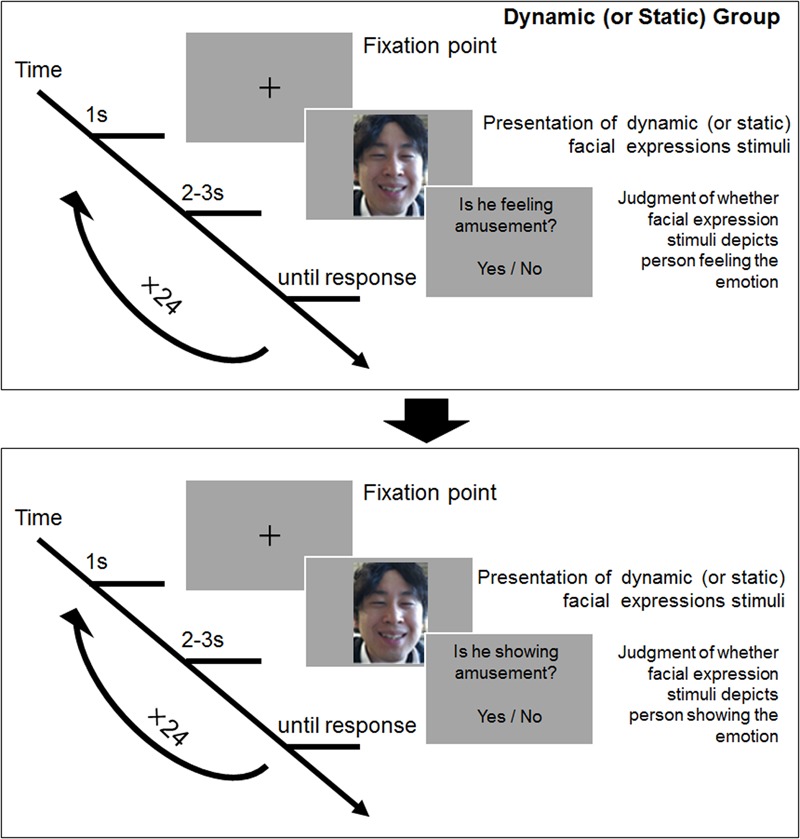
A depiction of the experimental flow for each trial.

The procedure of experimental tasks was conducted in line with the design implemented by McLellan et al. (2010). The task program was created using Visual C#. Each facial clip was presented on the screen of a laptop computer. Two groups of participants were assigned a facial stimuli presentation style: dynamic or static. The task program presented each trial into a block by culling the stimulus to be presented from a pool of 24 dynamic facial stimuli and 24 posed facial stimuli. We asked participants to perform two types of judgment tasks for the perception of emotional states via facial displays. The first was a *show condition* to judge whether the specific emotion was being depicted (e.g., “Is he showing sadness?”), and the second was a *feel condition* to judge whether the specific emotion was being experienced by the target (e.g., “Is she feeling happiness?”). Participants gave a yes-or-no answer to sort the show and feel conditions. The order of facial stimuli was randomized, and the blocks for the show and feel conditions were counterbalanced using a Latin Square design. **Figure 1** depicts the experimental flow.

Upon arrival at the laboratory and before doing the experimental tasks, participants were given careful instructions about the concept of genuine and posed facial displays and their requirements as participants. The instructions were as follows: “People sometimes express genuine facial displays caused by actual emotional experiences, while some people can express posed facial displays of emotion by intentional manipulation. In this study, we aim to understand whether people have the ability to detect these two types expressions accurately or not. There are two tasks we would like you to do. The first is to decide whether or not the expressions presented to you are showing each emotion, and the second is to decide whether or not the person depicted is feeling each emotion.”

After completing the instructions, all participants did a practice trial with facial stimuli not used in the main trial (semi-spontaneous anger, fear and posed anger, fear and a neutral stimulus). The facial stimuli for this rehearsal were made by a research assistant who was unaffiliated with the study. When participants completed the practice trial, the research assistant confirmed that participants understood the task. If there were no problems, the main trial was initiated. However, if there were issues understanding the task, participants were reminded of the instructions and asked to practice the trial again.

### Statistical Analysis

Although McLellan et al. (2010) conducted two analyses for the sensitivity between genuine, posed, and neutral facial displays utilizing only stimuli of posed displays, our study focused only on the comparison between genuine and posed displays as the target phenomenon for experiment, as well as for the sake of clarity. Yes-or-no answers to the facial displays were analyzed using a signal detection method that allows for separate modeling of the sensitivity and response criterion. Additionally, population-level sensitivity and the response criterion were estimated using a Bayesian hierarchical model (Rouder et al., 2007; Vuorre, 2017). In the vein of a generalized linear mixed model (Wright and London, 2009), our model (including a predictor) can be described as follows:

$$\begin{array}{c} y_{\text{ij}}\sim \text{Bernoulli}(p_{\text{ij}})\\ \Phi \text{​}\left(p_{ij}\right)=B_{0j}+B_{1j}*{\text{Display}}_{ij}+B_{2}*{\text{Presentation}}_{ij}\\ +B_{3}*{\text{Display}}_{ij}*{\text{Presentation}}_{ij} \end{array}$$

The outcomes *y*_ij_ were 1 if participant *j* responded “Yes” on trial *i*, and 0 if they responded “No”. Also, the outcomes for participant *j* and trial *i* were Bernoulli distributed with probability *p*_ij_. The probability was transformed into z-scores with Φ which represented the cumulative normal density function. *B*_0_ described the response criterion that corresponded to the tendency to answer “Yes” or “No”, and *B*_1_ described the sensitivity to facial displays. *B*_2_ described the difference in response criterion between dynamic and static presentations, and *B*_3_ described sensitivity. The sensitivity of the feel condition could be interpreted as the discriminability of emotional experiences in facial displays. Also, due to the assumed shortage of signal to be detected, *B*_1_in the show condition could be interpreted as the frequency of emotional concept recognition for genuine versus posed facial displays. To estimate the population-levels parameters for *B*_0_ and *B*_1_, multivariate normal distribution with means and a covariance matrix for the parameters are described in the following expression:

$$\left[\begin{array}{c} B_{0j}\\ B_{1j} \end{array}\right]\;\sim \;N(\left[\begin{array}{c} \mu_{0}\\ \mu_{1} \end{array}\right],\;\Sigma )$$

The means μ_1_ and μ_2_ can be interpreted as the population levels response criterion and sensitivity, respectively. In the following results, analysis was performed in R (3.3.3, R Core Team, 2016) using the *brms* packages (Bürkner, 2017). All iterations were set to 2,000 and burn in samples were set to 1,000, with the number of chains set to four. The value of Rhat for all parameters equalled 1.0, indicating convergence across the four chains.

## Results

**Table 1** shows the percentage of Yes responses by judgment condition, presentation style, and facial displays for all emotions in total, as well as separated by each emotion. The following results were expected to be found according to our hypotheses: (1) genuine displays would be aligned with an answer of “Yes” in both the show and feel conditions, (2) posed displays would be answered with “Yes” for the show condition, but not the feel condition, and (3) neutral displays would be responded with “No” in both conditions. Comparisons using **Table 1** indicated several observations. For example, static presentations decreased the percentage of Yes responses in the show condition for all emotions. In the case of the feel condition, dynamic presentation promoted discriminability for all emotions. Hierarchical signal detection theory was applied in order to confirm these observations. Although results for the response criteria were also estimated, only the results for the sensitivity to displays are reported below to avoid redundancy.

**Table 1:** List of the percentage of Yes responses that emerged in judgment conditions and facial displays.

	Show condition (% yes)		Feel condition (% yes)	
Display type	Dynamic	Static	Dynamic	Static
**All emotions**				
Neutral	2	12	10	17
Posed	88	78	31	59
Genuine	78	70	75	66
**Amusement**				
Neutral	0	2	0	0
Posed	98	91	38	80
Genuine	95	95	85	86
**Surprise**				
Neutral	3	2	7	2
Posed	95	70	23	47
Genuine	73	46	75	52
**Disgust**				
Neutral	5	32	13	46
Posed	90	86	32	75
Genuine	97	96	75	66
**Sadness**				
Neutral	0	13	18	18
Posed	67	66	32	34
Genuine	48	43	63	59

### The Show Condition Path to All Emotions

**FIGURE 2:**
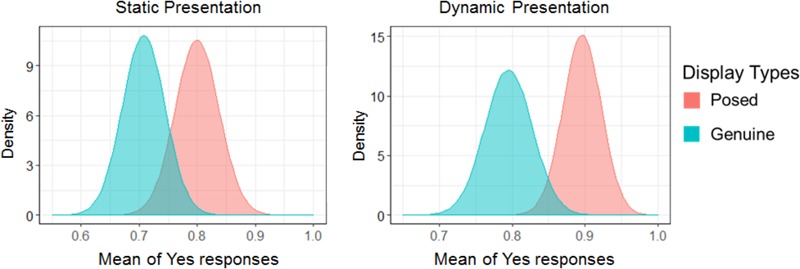
The mean of Yes responses in the show condition for all emotions. The distance between the two distributions can be interpreted as the discriminability of facial displays.

**Figure 2** describes the percentage of Yes responses in the show condition by the type of facial displays for all emotions and presentation styles. Furthermore, results of a hierarchical signal detection method to estimate parameters for the show condition can be seen in **Table 2**. If the 95% credible interval of the parameters does not include zero, it can be inferred that there is a significant effect as in classical statistical hypothesis testing. **Table 2** shows that a negative value for the sensitivity to displays emerged, which indicates that participants responded “Yes” more frequently to posed displays than genuine displays (β_1_ = -0.37 [-0.59, -0.16]). In other words, participants were able to differentiate genuine facial displays from posed ones. Specifically, participants judged posed displays as the facial display showing a specific target emotion more frequently than the genuine displays.

**Table 2:** Estimated parameters on each condition for all emotions using a signal detection model.

Parameter	MAP	95%CI[]
**Show condition**		
Response criteria (Beta1)	1.06	[0.86, 1.25]
Sensitivity to display (Beta2)	-0.38	[-0.57, -0.16]
Response criteria between presentations (Beta3)	0.46	[0.07, 0.82]
Sensitivity to display between presentations (Beta4)	-0.13	[-0.56, 0.25]
**Feel condition**		
Response criteria (Beta1)	-0.12	[-0.25, -0.01]
Sensitivity to display (Beta2)	0.66	[0.50, 0.85]
Response Criteria between presentations (Beta3)	-0.72	[-0.99, -0.49]
Sensitivity to display between presentations (Beta4)	1.03	[0.64, 1.35]

*MAP stands for Maximum a Posteriori estimate. 95% CI represents 95% credible intervals*.

### The Feel Condition Path to All Emotions

**FIGURE 3:**
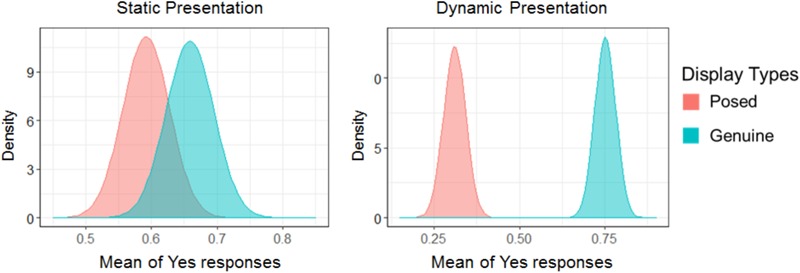
The mean of Yes responses in the feel condition for all emotions. The distance between the two distributions can be interpreted as the discriminability of facial displays.

The percentage of Yes responses for the feel condition to all emotions is presented in **Figure 3**. Also, **Table 2** provides estimated parameters for the feel condition. The results for the sensitivity to displays indicated that genuine displays cause Yes responses on the feel condition to occur more frequently than posed ones (*B*_1_ = 0.68 [0.49, 0.85]). Moreover, the results for the sensitivity to displays between presentation styles indicated that when the presentations style was dynamic, the sensitivity to differentiate between genuine and posed ones was higher than when it was static (*B*_3_ = 0.98 [0.63, 1.34]). Taken together, perceivers could distinguish genuine from posed facial expressions and their sensitivity was higher under the conditions that facial displays were presented dynamically, rather than statically.

### The Show Condition Across Emotions

**FIGURE 4:**
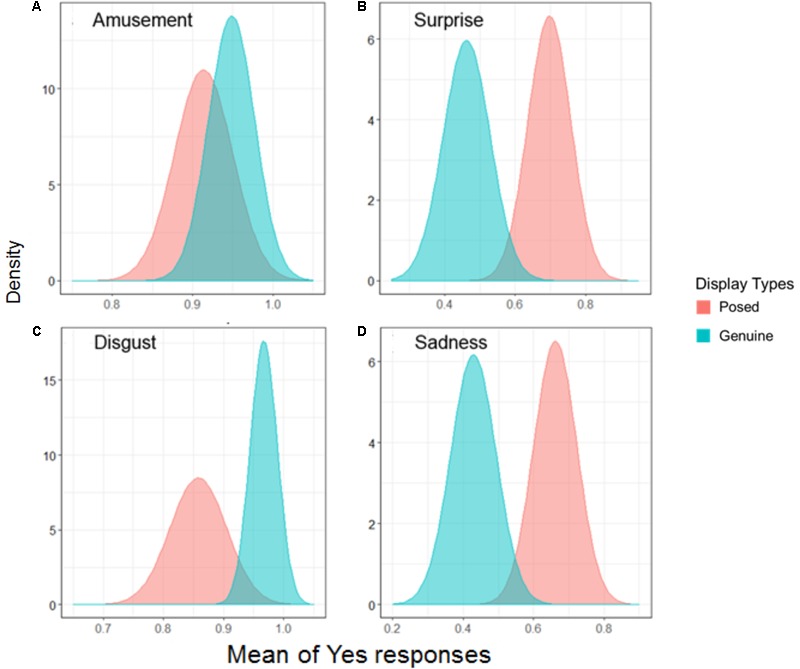
The mean of Yes responses on the show condition across each emotion. The distance between the two distributions can be interpreted as the discriminability of facial displays. Comparisons are shown for each specific emotion: **(A)** amusement, **(B)** surprise, **(C)** disgust, and **(D)** sadness.

Next, to consider the specific characteristics across different types of emotions, we investigated data from the show condition for each emotion. **Figure 4** shows the percentage of Yes responses in the show condition across emotions. In this case, we conducted a simple signal detection model that did not include a hierarchical structure to avoid model complexity and to stabilize the convergence. The estimated parameters are described in **Table 3**. For amusement, a result for the sensitivity was not found. For surprise, the value of the sensitivity to displays was negative (β_1_ = -0.78 [-1.25, -0.44]). The results of sadness indicated that the value of the sensitivity to displays was negative (*B*_1_ = -0.53 [-0.86, -0.21]). For disgust, the results indicated that the value of the sensitivity to displays was positive (*B*_1_ = 0.69 [0.19, 1.24]). In sum, posed displays of surprise and sadness were consistent with the results for all emotions, but disgust was found to be in the opposite direction for the showing condition.

**Table 3:** Estimated parameters on show condition across each emotion using a Bayesian signal detection model.

Parameters	MAP	95%CI[]
**Amusement**		
Response criteria (Beta1)	1.73	[1.38, 2.36]
Sensitivity to display (Beta2)	-0.10	[-0.80, 0.48]
Response criteria between presentations (Beta3)	0.81	[0.01, 1.94]
Sensitivity to display between presentations (Beta4)	-0.80	[-2.15, 0.29]
**Surprise**		
Response criteria (Beta1)	1.10	[0.79, 1.44]
Sensitivity to display (Beta2)	-0.78	[-1.25, -0.44]
Response criteria between presentations (Beta3)	1.15	[0.55, 1.84]
Sensitivity to display between presentations (Beta4)	-0.34	[-1.25, 0.30]
**Disgust**		
Response criteria (Beta1)	1.18	[0.90, 1.49]
Sensitivity to display (Beta2)	0.69	[0.19, 1.24]
Response criteria between presentations (Beta3)	0.13	[-0.39, 0.82]
Sensitivity to display between presentations (Beta4)	-0.13	[-1.34, 0.86]
**Sadness**		
Response criteria (Beta1)	0.43	[0.19, 0.65]
Sensitivity to display (Beta2)	-0.53	[-0.86, -0.21]
Response criteria between presentations (Beta3)	0.06	[-0.46, 0.50]
Sensitivity to display between presentations (Beta4)	0.13	[-0.55, 0.78]

*MAP stands for Maximum a Posteriori estimate. 95% CI represents 95% credible intervals*.

### The Feel Condition Across Emotions

**FIGURE 5:**
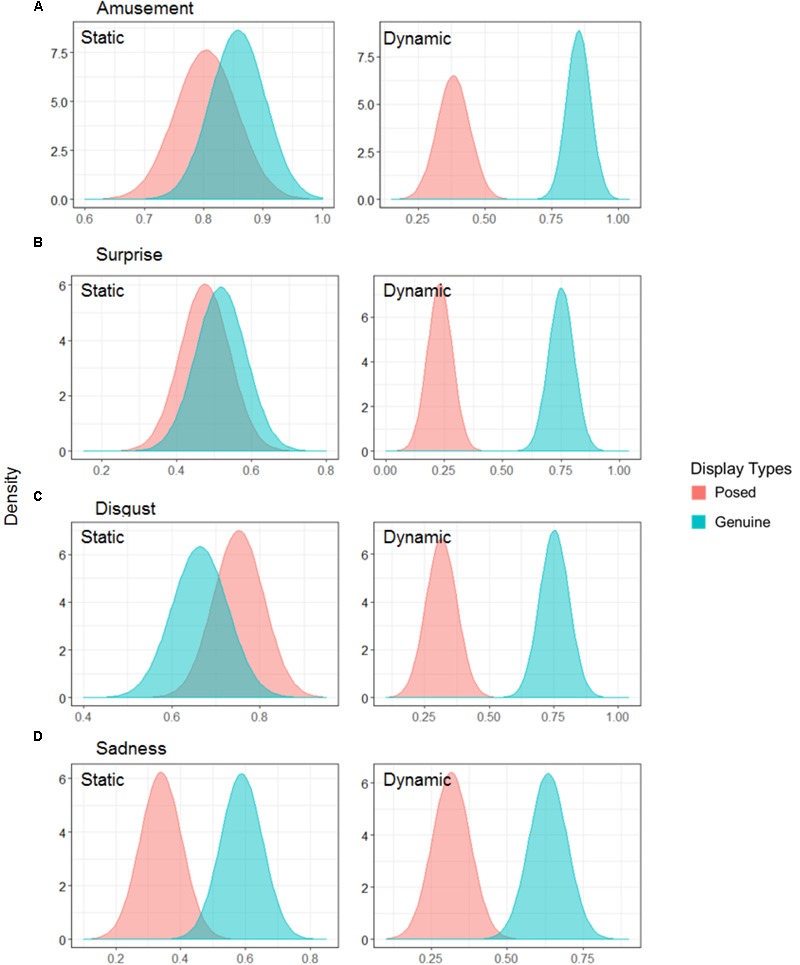
The mean of the Yes response on the feel condition across each emotion. The distance between the two distributions can be interpreted as the discriminability of facial displays. Comparisons are shown for each specific emotion: **(A)** amusement, **(B)** surprise, **(C)** disgust, and **(D)** sadness.

Finally, we provided estimated parameters using data on the feel condition across emotions. **Figure 5** shows the marginal effects on the feel condition across emotions, and **Table 4** lists the estimated parameters. For amusement, the result for the sensitivity indicated the same directions as the parameters for the feel condition and all emotions (*B*_1_ = 0.80 [0.42, 1.15]; *B*_3_ = 1.13 [0.39, 1.87]). For surprise, the results were consistent with the parameters in the path to all emotions (*B*_1_ = 0.80 [0.41, 1.09];*B*_3_ = 1.30 [0.61, 1.96]). For disgust, the results indicated that the values of the two types of sensitivity to displays were positive (*B*_1_ = 0.45 [0.12, 0.78]; *B*_3_ = 1.40 [0.71, 2.08]). The results for sadness indicated that the sensitivity to displays was positive (*B*_1_ = 0.72 [0.41, 1.06]). Subsequently, all results across emotions found that participants judged the genuine displays as the facial display where the person on-screen was experiencing the specific target emotion, rather than posed displays. Furthermore, when participants differentiated the genuine and posed facial displays in terms of the existence of emotional experiences for amusement, surprise, and disgust, dynamic presentations notably increased the sensitivity to displays compared to static ones.

**Table 4:** Estimated parameters on feel condition across each emotion using a Bayesian signal detection model.

Parameters	MAP	95%CI[]
**Amusement**		
Response criteria (Beta1)	0.26	[0.04, 0.52]
Sensitivity to display (Beta2)	0.80	[0.42, 1.15]
Response criteria between presentations (Beta3)	-1.16	[-1.66, -0.66]
Sensitivity to display between presentations (Beta4)	1.13	[0.39, 1.87]
**Surprise**		
Response criteria (Beta1)	-0.40	[-0.64, -0.15]
Sensitivity to display (Beta2)	0.80	[0.41, 1.09]
Response criteria between presentations (Beta3)	-0.68	[-1.14, -0.19]
Sensitivity to display between presentations (Beta4)	1.30	[0.61, 1.96]
**Disgust**		
Response criteria (Beta1)	0.10	[-0.14, 0.34]
Sensitivity to display (Beta2)	0.42	[0.12, 0.78]
Response criteria between presentations (Beta3)	-1.16	[-1.64, -0.65]
Sensitivity to display between presentations (Beta4)	1.42	[0.71, 2.08]
**Sadness**		
Response criteria (Beta1)	-0.46	[-0.69, -0.20]
Sensitivity to display (Beta2)	0.72	[0.41, 1.06]
Response criteria between presentations (Beta3)	-0.08	[-0.54, 0.42]
Sensitivity to display between presentations (Beta4)	0.16	[-0.48, 0.84]

*MAP stands for Maximum a Posteriori estimate. 95% CI represents 95% credible intervals*.

## Discussion

The present study investigated whether or not people can distinguish between genuine and posed facial displays of emotion by focusing on dynamic or static presentation styles. The results indicated three key findings. First, people judged posed displays as showing surprise and sadness more than the genuine displays. Second, the results of the feel condition disambiguated that people distinguish between genuine and posed facial displays of emotion in terms of their estimation that the experiences were authentically felt. Finally, the study found that perceivers are more capable of differentiating whether expressers are having a felt emotional experience when dynamic facial display processes are present over static ones.

### Judging Whether the Specific Emotion Was Being Shown

This study clarified the characteristics of genuine and posed displays, with the latter being recognized as the facial display showing a specific target emotion (described in **Figure 2**). This result is consistent with several previous studies in which the percentages of observers matching the predicted emotion to posed facial displays were considerably higher than spontaneous ones (e.g., Motley and Camden, 1988; Naab and Russell, 2007; Calvo and Nummenmaa, 2016). This result suggests that posed facial expressions are vital to the process of conveying an emotion, but that their utility does not manifest itself evenly for all emotions. For amusement, there were no differences between spontaneous and posed displays when it came to whether the target emotion was being shown. Motley and Camden (1988) suggested that only spontaneous facial expressions of positive emotions and not negative ones were recognizable above chance level, as is similar to the recognition of posed faces. In this case, it could be suggested that the perceptual information used to show amusement is not different between spontaneous and posed displays. For disgust, the results of the present study indicated that when judging the show condition for a target emotion genuine displays did so more frequently than posed displays, as described in **Figure 4**. Facial expressions of disgust function to convey potential threats like biological factors directly linked to death to an interlocutor (Tybur et al., 2013), and it is therefore possible that spontaneous expressions might contain the perceptual information to convey disgust more clearly than posed expressions.

### Judging Whether the Specific Emotion Was Being Felt

The current study revealed that perceivers possess a sensitivity to facial displays that is related to the accurate inference of the emotional experiences from genuine, but not posed, facial expressions. As shown in section “The Feel Condition Across Emotions,” this study observed no difference in this discriminability across emotions. Considering that there was a difference among emotions in show condition, this result is impressive. The ability to detect emotional experiences in facial expressions might be more important or more general for successful social interactions than the ability to detect the mere showing of an emotion. Both genuine and posed facial expressions can be regarded as means to express the internal state of the person signaling, that in turn directs the behavior of the observer, establishes a representation of the world for the expresser to draw from, and allows them to commit to future courses of action (Scarantino, 2017; Van Kleef, 2017). The difference between the two expressions is the endogenous nature of emotional experiences, which can be connected to the trustworthiness of the message in facial displays. From the perspective of the biological and evolutionary function of social emotions, people respond sensitively to signals with high credibility and emotional salience (Niedenthal and Brauer, 2012). Therefore, the results of this study extend the literature from previous studies consistent with the hypothesis that people can discern genuine and posed facial displays (McLellan et al., 2010; Dawel et al., 2015). However, there are small differences between previous findings and our results. Previous studies suggested that the sensitivity for emotional experiences to facial displays was specific across each emotion rather than a generalized skill, but we found that specificity for the types of emotion disappeared when non-social spontaneous facial expressions were used as genuine facial stimuli. Therefore, our results offer evidence that people might have a general discriminability that allows them to differentiate between genuine and posed displays when it comes to perceiving felt emotional content in an expresser. Moreover, the facial stimuli presented in this study were morphologically distinct between genuine and posed facial displays, as suggested by Namba et al. (2017c). The accurate inference of emotional experience may be due to differences in morphological features, but not intensity.

### Dynamic Information Related to the Sensitivity to Facial Displays

Interestingly, the signal detection model in the present study provides empirical support for the idea that sensitivity for the perception of emotional experiences to displays depends upon whether the presentation style is dynamic or static. As suggested in previous studies, this finding indicates that dynamic facial displays simply offer more information for a perceiver to parse the emotional experience of the expresser (Krumhuber and Manstead, 2009; Krumhuber et al., 2013), due to a tradeoff in the amount of information available in dynamic interactions as compared to static interactions. Ambadar et al. (2005) also showed the advantage of dynamic presentation in an emotion recognition task as one that captures the intrinsic temporal quality of an unfolding expression rather than mere increases in static facial frames. Our study did not compare dynamic and multi-static stimuli, but did show that non-linear motion of spontaneous expressions might raise ecological validity, and suggested that such situations could increase the discriminability of the expresser’s experiences of emotions like surprise and disgust. Our findings could also imply that further research related to the perception of emotional experiences in facial expressions, such as those in the realm of emotional contagion (Hatfield et al., 2014), might benefit from using dynamic genuine facial expressions as stimuli because the standardized practice of presenting static stimuli may play a role in the lack of detection of emotional experiences from facial displays.

### Limitations and Future Studies

While this study showed that people can distinguish between genuine and posed facial displays of emotion and that this sensitivity depends on whether the facial displays unfolded dynamically or not, several limitations should be noted. First, a signal detection model using binary reactions allowed for the provision of response criteria in addition to sensitivity. However, Dawel et al. (2017) indicated that the yes-or-no response provides far less information than a rating scale about the relative perceived genuineness of different stimuli. Therefore, additional studies should be conducted using rating scales, such as a neutral midpoint scale (e.g., perceived genuineness: -7 = *completely fake*; 0 = *don’t know*; +7 = *completely genuine*, Dawel et al., 2017).

Next, the results of this study should be interpreted for only the four emotions investigated: amusement, surprise, disgust, and sadness. Although fearful displays have typically been used in previous studies (McLellan et al., 2010; Douglas et al., 2012; Dawel et al., 2015), the current study did not examine fear due to the lack of evidence in the domain of spontaneous facial expressions of fear. Also, other emotions such as happiness (McLellan et al., 2010), anger and contempt (Fischer and Roseman, 2007) should be considered to extend the evidence base of these findings to future studies.

In addition, while a Bayesian probit regression procedure-based signal detection model was able to produce these results, a larger sample size of study participants and facial stimuli could provide for a more robust understanding of the effects and allow for separate analyses of each emotion of interest through a generalized linear model. The data from the present study will be appended as Supplementary Material so that researchers can access it as open data and further examine or build upon the evidence base in future collaborative research projects or novel statistical approaches.

Finally, we used spontaneous facial expressions that were secretly recorded to avoid the effects of intentional manipulation. Although these facial stimuli can allow for fine-grained understanding of the sensitivity to facial displays to be explored, such stimuli cannot control other subtle non-verbal cues like head or eye movements. We avoided stimuli that included these features as much as possible during the facial stimuli selection stage, but it is difficult to control for these subtle actions in a non-social experimental environment designed to capture genuine facial stimuli. To overcome these barriers, further studies might consider the use of computerized facial expressions (Jack et al., 2014; Krumhuber and Scherer, 2016), as it may be possible to conduct research controlling small movements in facial stimuli by letting an avatar load the genuine displays.

## Conclusion

The current study revealed that people are capable of distinguishing genuine from posed facial expressions by judging whether the target emotion was being shown and felt by the expresser. Specifically, posed displays were more frequently judged as the facial expressions showing specific emotions of surprise and sadness than genuine displays, whereas genuine displays were evaluated as the felt expressions of a target emotion in the case of amusement, surprise, disgust and sadness. Additionally, variability in the discriminability of authentic experiences was examined and found to depend on whether the facial display was dynamically or statically presented. The sensitivity to detect emotional experiences of amusement, surprise, and disgust was lower in the statically presented facial expressions, whereas dynamic information enhanced the discriminability for observers to detect the emotional experiences of others depicted in facial displays. Still, as the perception of facial expressions depends heavily on the surrounding context, it will be necessary to corroborate these findings with data from many other investigations. We hope that these distinctions on the type of stimuli presented and their characteristics can be taken into consideration by future researchers interested in the domain of emotional facial expressions and their properties.

## Ethics Statement

All procedures performed in studies involving human participants were in accordance with the ethical standards of the institutional and national research committee.

## Author Contributions

SN conducted the research, statistical analysis, and served as the primary author of the manuscript. RK offered revisions, summary, and literature review. MM and TN contributed to confirmation of the research protocol, further review of methods and analysis, and feedback on the manuscript.

## Conflict of Interest Statement

The authors declare that the research was conducted in the absence of any commercial or financial relationships that could be construed as a potential conflict of interest.
